# Benefit finding in individuals undergoing maintenance hemodialysis in Shanghai: a latent profile analysis

**DOI:** 10.3389/fpsyg.2024.1292175

**Published:** 2024-03-04

**Authors:** Jie Yang, Yong-qi Li, Yan-lin Gong, Hong-li Yan, Jing Chen, Ling-ling Liu, Jing Wu, Jing Chu

**Affiliations:** ^1^School of Nursing, Naval Medical University, Shanghai, China; ^2^School of Health Services Management, Southern Medical University, Guangzhou, China; ^3^Department of Nephrology, Shanghai Chang Zheng Hospital, Shanghai, China

**Keywords:** maintenance hemodialysis, benefit finding, latent profile analysis, social support, coping style

## Abstract

**Objective:**

This multi-center cross-sectional study aimed to delineate latent profiles of benefit finding (BF) in individuals undergoing maintenance hemodialysis (MHD) in Shanghai and examine associations between these BF profiles, social support, and coping style.

**Methods:**

A total of 384 individuals undergoing MHD (mean age = 57.90, SD = 13.36) were assessed using the Benefit Finding Scale, Simplified Coping Style Questionnaire, and Perceived Social Support Scale. Latent profile analysis (LPA) identified distinct BF categories. Analysis of variance (ANOVA) evaluated the correlation between BF groups and demographic variables, while the relationship between BF, social support, and coping style was tested through correlation and multiple regression analyses.

**Results:**

LPA identified three BF groups: rich BF (54.17%), moderate BF (41.14%), and poor BF (4.69%). Regression analyses indicated that positive coping and social support are protective factors for BF. Additionally, older age and heightened understanding of MHD correlated with higher BF levels.

**Conclusion:**

The findings highlighted the importance of recognizing different BF profiles in individuals on MHD and working toward promoting BF levels in the rich BF and moderate BF groups, while helping the poor BF group to identify and address their challenges. Medical professionals should consider interventions tailored to individual psychological profiles to improve mental health and quality of life outcomes in this population.

## 1 Introduction

Chronic kidney disease (CKD) has emerged as a serious global public health problem, marked by its high prevalence and increasing burden (Cockwell and Fisher, 2020). Globally, the 2017 prevalence of CKD was 9.1% (95% UI: 8.5–9.8), and mortality rates from CKD increased by 41.5% compared to 1990 (GBD Chronic Kidney Disease Collaboration, 2020). As CKD progresses, individuals often experience a decline in quality of life and face socioeconomic challenges (Webster et al., 2017). Maintenance hemodialysis (MHD) is the primary renal replacement therapy for individuals with kidney failure (Thurlow et al., 2021). Notably, China has one of the highest populations undergoing MHD. According to the Chinese National Renal Data System (CNRDS), the number of registered individuals on MHD in China reached 749,573 by the end of 2021 (Kao et al., 2023). This lifelong chronic condition not only affects the quality of life but also imposes substantial burdens on families and society (GBD Chronic Kidney Disease Collaboration, 2020), often leading to negative emotional states such as sadness, pain, anxiety, and depression (Pop-Jordanova and Polenakovic, 2013). A meta-analysis including 2,822 individuals undergoing MHD across 27 studies found an estimated proportion of 62% (0.54 ~ 0.71) prevalence of depressive symptoms with a peak incidence of 95% (0.88–1.02) (Ravaghi et al., 2017), significantly higher than the general population (Tian et al., 2021).

With the advent of positive psychology, research has identified that some individuals may experience positive psychological changes, termed benefit finding (BF), in response to illness (Sun et al., 2022; Li et al., 2023). BF, indicative of cognitive and behavioral adaptation to adverse events (Helgeson et al., 2006), can encompass a profound sense of personal strength, greater appreciation for life, enhanced relationships, spiritual growth, and new life opportunities (Aspinwall and Tedeschi, 2010). BF is also considered an indicator of mental health (Yan et al., 2023). Studies have shown that higher levels of BF in individuals correlate with greater happiness and fewer negative emotions during illness (Wepf et al., 2022). BF in individuals undergoing MHD reflects their ability to identify positive aspects following dialysis, leading to a transformation of this challenging experience into one with meaningful implications (Aspinwall and Tedeschi, 2010). BF has been studied in patients and caregivers in the context of cancer and diabetes (Tran et al., 2011; Lassmann et al., 2021; Zimmaro et al., 2021; Li et al., 2023; Yan et al., 2023), identifying influencing factors ranging from demographic (gender, age, education level, type of work, and marital status), disease-specific (cause of illness, duration of illness) (Zimmaro et al., 2021; Zhu et al., 2022; Mei et al., 2023), and psychosocial factors (social support and coping styles) (Qiu et al., 2022; Li et al., 2023).

Previous studies on BF conducted by Jue et al. (2023) and Dan-li and Wen-huan (2022), comprising a total of 861 individuals undergoing MHD, revealed that BF levels in this population were lower than in other chronic disease populations. These studies also highlighted the influence on BF levels of multiple factors including age, education, occupation, and duration of dialysis. While these research efforts have enriched our understanding of BF in the individuals on MHD, they have overlooked the heterogeneity of BF across different populations or regions, attributable to the unique characteristics of the study participants and varied research methods. Traditional research methods, focusing on the total or average questionnaire scores, are effective in elucidating correlations between variables. However, these methods fall short in comparing differences across various categories of variables. In response to this limitation, recent research in diverse fields, including education, sociology, and psychology, has increasingly adopted latent profile analysis (LPA). LPA is utilized to identify latent categorizations of individuals based on their distinct response patterns to a range of apparent variables, thereby understanding the proportion of different categories within the population. This approach not only maximizes inter-category differences and minimizes intra-category variations but also enhances the accuracy and validity of categorizations through objective statistical indicators (Wen et al., 2023).

The Pearlin stress process model, which provides a comprehensive framework for understanding the influence of stressors on health outcomes, distinguishes between three core elements, namely, the sources of stress, the mediators of stress, and the manifestations of stress (Pearlin et al., 1981). For individuals with kidney failure undergoing MHD, the experience of making treatment decisions (Saeed et al., 2020) and enduring chronic strains from various factors such as complications, self-management, treatment-life balance, and financial burdens are common sources of stress (de Vries et al., 2021; Tao et al., 2023). Throughout their treatment, individuals on MHD actively seek external benefits, continually garner social support, and employ positive coping strategies to mitigate the influence of these stressors on their physical, mental, and overall well-being. Although everyday stress may not invariably harm individuals and can be adaptive (Thoits, 2010), BF, in this context, is a manifestation of a positive response to psychological stress. The stress process model has garnered substantial empirical support and has been applied across a wide range of samples (Yu et al., 2020). However, the application of this model to understanding the role of BF in individuals on MHD has not been previously explored.

Therefore, grounded in the stress process model, and adopting a positive psychology perspective, this study aims to accurately characterize BF in individuals on MHD. This investigation will lay a foundation for enhancing health-related behaviors and improving the quality of life outcomes in this clinical group. The primary objective is to elucidate the heterogeneity of BF among individuals on MHD in Shanghai, China, through LPA, identifying distinct BF subgroups and analyzing the status and characteristics. The secondary objectives include investigating the relationship between BF and social support and coping styles and exploring the influence of demographic, disease-related, and psychosocial factors on BF latent profiles. This comprehensive approach will enable the development of tailored intervention strategies for different BF subgroups, ensuring targeted and effective support for individuals on MHD.

## 2 Methods

### 2.1 Participants

Individuals on MHD at four hospitals in Shanghai between June and December 2022 were selected utilizing a convenience sampling method (Table 1).

**Table 1:** The criteria of the subjects.

Inclusion criteria	Exclusion criteria
Individuals undergoing MHD for kidney failure. Aged ≥18 years. Capable of engaging in a conscious conversation. Normal hearing and cognitive capacity.	Critically ill individuals. Presence of a functional or organic mental illness. Inability to cooperate with the investigator.

A total of 396 questionnaires were distributed. We excluded 12 incompletely completed questionnaires, thereby yielding a total of 384 that were included in the final analysis. The cohort included 226 male individuals and 158 female individuals, with a mean age of 57.90 ± 13.36 years. Detailed demographic information for this study cohort is shown in Table 2.

**Table 2:** Demographic characteristics of participants (*n* = 384).

Categorical variable	*n* (%)
**Gender**	
Male	226 (58.85)
Female	158 (41.15)
**Age (years)**	
<45	78 (20.31)
45–60	121 (31.51)
>60	185 (48.18)
**Marital status**	
Married	305 (79.43)
Unmarried	42 (10.94)
Divorced	17 (4.43)
Widowed	20 (5.21)
**Education**	
Primary or lower	11 (2.86)
Junior high	105 (27.34)
Senior high	132 (34.38)
College or higher	136 (35.42)
**Occupation**	
Student	1 (0.26)
Full-time	78 (20.31)
Part-time	14 (3.65)
Retire	246 (64.06)
Unemployment	45 (11.72)
**Economic burden**	
Very mild	48 (12.50)
Mild	104 (27.08)
Moderate	161 (41.93)
Serious	56 (14.58)
Very serious	15 (3.91)
**Number of chronic diseases**	
0	75 (19.53)
1	143 (37.24)
2	103 (26.82)
3	41 (10.68)
4	12 (3.13)
≥5	10 (2.60)
**Duration of MHD**	
0–1 year (including 1 year)	73 (19.01)
1–5 years (including 5 years)	134 (34.90)
5–10 years (including 10 years)	82 (21.35)
10–20 years (including 20 years)	74 (19.27)
>20 years	21 (5.47)
**Kidney transplantation**	
Yes	31 (8.07)
No	353 (91.93)
**Understanding degree of MHD**	
No understanding	55 (14.32)
Partial understanding	143 (37.24)
Adequate understanding	154 (40.10)
Complete understanding	32 (8.33)
**Self-assessed MHD severity**	
Very mild	29 (7.55)
Mild	128 (33.33)
Moderate	221 (57.55)
Serious	4 (1.04)
Very serious	2 (0.52)
**Self-assessed life impact**	
Very mild	4 (1.04)
Mild	48 (12.50)
Moderate	195 (50.78)
Serious	122 (31.77)
Very serious	15 (3.91)

### 2.2 Measures

#### 2.2.1 General information questionnaire

A self-designed general information questionnaire collected general demographic data, including gender, age, marital status, education, occupation, and economic burden. Additionally, this questionnaire gathered disease-related information, encompassing the number of chronic diseases, duration of MHD, self-assessed severity and life impact of MHD, and the individual’s degree of understanding of MHD-related knowledge.

#### 2.2.2 Benefit finding scale

The scale comprises 26 items across six dimensions, each rated on a 5-point Likert scale ranging from 0 (“not at all”) to 4 (“extremely”). Higher aggregate scores signify a higher level of BF (Yan et al., 2023). In this study, the overall reliability of the scale, as indicated by Cronbach’s α coefficient, was 0.935. Cronbach’s α coefficient values for individual dimensions were as follows: 0.774 for *spiritual growth*, 0.856 for *appreciation of living and life*, 0.703 for *awareness of social support*, 0.879 for *personal growth*, 0.925 for *altruistic behavior*, and 0.836 for *health behavior changes*.

#### 2.2.3 Simplified coping style questionnaire

This questionnaire consists of 20 items across two dimensions: *positive coping* and *negative coping* (Huang et al., 2021). Responses to each item are measured on a 4-point Likert scale ranging from 0 to 3, with higher total scores indicating a predominant use of this coping style. In this study, Cronbach’s α coefficient for the Simplified Coping Style Questionnaire (SCSQ) was 0.844, with positive coping at 0.854 and negative coping at 0.712. The SCSQ is a widely utilized instrument in Chinese clinical settings.

#### 2.2.4 Perceived social support scale

The Perceived Social Support Scale (PSSS), used to assess perceived social support from various sources (Peng et al., 2023), including 12 items categorized into three dimensions, namely family support, friend support, and other support. Each item is rated on a 7-point Likert scale, ranging from 1 (“strongly disagree”) to 7 (“strongly agree”). Higher aggregate scores indicate significantly perceived support. In the present study, overall Cronbach’s α for the scale was 0.877, with the dimensions of *family support*, *friend support*, and *other support* scoring 0.854, 0.898, and 0.883, respectively.

### 2.3 Data collection

Prior to conducting the survey, approval was sought and obtained from the administrators of the hemodialysis unit of hospital. Informed consent was obtained from all participants before the questionnaires were distributed. Detailed explanations regarding the survey were provided by the researcher to ensure participants’ understanding. Upon completion, questionnaires were immediately collected by the researcher for review. In cases where responses were missing or incorrectly filled, participants were promptly consulted for verification and correction, thereby ensuring the accuracy and authenticity of the data collected. This study complied with the principles outlined in the Declaration of Helsinki for research involving human participants and was approved by the Committee on Ethics of Medicine, Naval Medical University, Shanghai, China.

### 2.4 Data analysis

EpiData 3.0 was used for data entry and organization. Subsequently, IBM SPSS 26.0 was employed for descriptive statistical analysis and multiple regression analyses. LPA was performed using Mplus 8.3. The criteria for determining the optimal model included as follows: lower Akaike Information Criterion (AIC), Bayesian information criterion (BIC), and sample-corrected Bayesian information criterion (aBIC) than competing models. Additionally, an entropy value of >0.8 and a significant Lo–Mendell–Rubin adjusted likelihood ratio test (LMR-LRT) were indicative of model adequacy. The questionnaire scale scores were summarized using mean and standard deviation. Differences among various demographic variables were assessed using analysis of variance (ANOVA) and chi-square tests, as appropriate. Ordered logistic regression analysis was used to explore the influencing factors of BF. The threshold for statistical significance is set at a *p*-value of 0.05.

## 3 Results

### 3.1 Common method deviation test

Harman’s single-factor test was applied to all original variables in this study. The analysis extracted 13 factors with eigenvalues greater than 1. The first factor accounted for 25.88% of the variance, which did not exceed 40% of the total variance explained. Thus, this study did not exhibit a significant common method bias.

### 3.2 Latent profile analysis of BF among individuals on MHD

Using six dimensions of BF as indices, five potential category models were evaluated. The results indicated decreasing AIC, BIC, and aBIC values with increasing numbers of latent profiles. All models had an entropy value of >0.8, with model 3 exhibiting the highest entropy. However, the LMR test demonstrated statistical significance only in model 3 (*p* > 0.05, for other models), leading to its selection as the most suitable for LPA. Detailed model fitting information is provided in Table 3.

**Table 3:** Fitting information for the latent profile analysis.

Model	AIC	BIC	aBIC	Entropy	LMR(*p*)	BLRT(*p*)	Probability of attribution
1	12590.289	12637.697	12599.622				
2	11997.330	12072.392	12012.108	0.842	0.0576	<0.001	105/279
3	**11728.121**	**11830.838**	**11748.343**	**0.891**	**<0.001**	**<0.001**	**158/18/208**
4	11650.604	11780.975	11676.27	0.821	0.2852	<0.001	62/139/13/170
5	11561.166	11719.192	11592.278	0.842	0.064	<0.001	13/65/117/48/141

The bold values represents model three was selected as a meaningful latent profile groups for this study.

### 3.3 Naming latent profiles of BF among individuals on MHD

**Figure 1:**
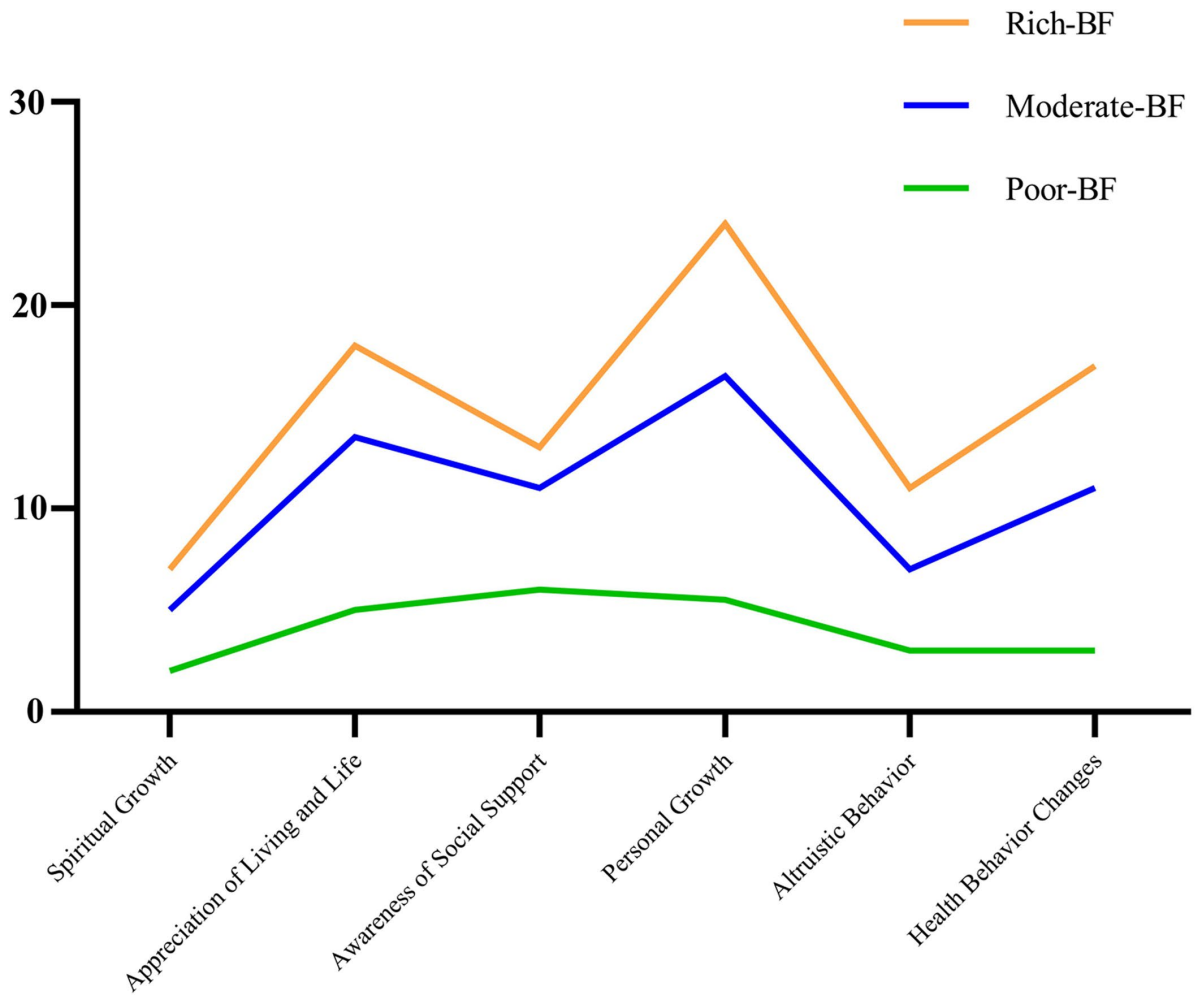
Conditional mean values of the three latent profiles on the six dimensions.

The comparative analysis of the three BF categories revealed a hierarchical pattern: group 1 (***rich BF***) with the highest scores (87.57 ± 8.07), group 2 (***moderate BF***) with medium scores (62.78 ± 8.18), and group 3 (***poor BF***) with the lowest scores (26.06 ± 10.69). Significant differences were observed in the total and dimensional scores among these groups (*p* < 0.05), as shown in Table 4 and Figure 1.

**Table 4:** Comparison of the BF scores of individuals on MHD with different latent profiles.

Group	*N*	BF scores (M ± SD)	Spiritual growth (terms 1–2)	Appreciation of living and life (terms 3–7)	Awareness of social support (terms 8–11)	Personal growth (term 12–18)	Altruistic behavior (terms 19–21)	Health behavior changes (terms 22–26)
1	208	87.57 ± 8.07	6.51 ± 1.86	17.94 ± 2.27	12.99 ± 2.52	23.74 ± 3.05	9.78 ± 2.74	16.60 ± 2.79
2	158	62.78 ± 8.18	4.91 ± 2.01	13.44 ± 3.21	10.41 ± 2.91	16.30 ± 3.30	6.91 ± 2.62	10.82 ± 3.19
3	18	26.06 ± 10.69	2.61 ± 2.19	5.78 ± 3.71	5.56 ± 2.95	5.72 ± 3.35	3.06 ± 2.61	3.33 ± 2.45
F		723.599	53.596	233.631	87.172	430.395	86.455	284.993
P		<0.001	<0.001	<0.001	<0.001	<0.001	<0.001	<0.001

### 3.4 Univariate analysis of latent profiles of BF among individuals on MHD

#### 3.4.1 Influences of demographic variables on the latent profile

Comparisons between the BF scores and demographic variables across groups exhibited significant differences in age, marital status, number of chronic diseases, understanding of MHD, and self-assessed MHD severity (*p* < 0.05), as shown in Table 5.

**Table 5:** Influences of demographic variables on the latent profile.

Variable	Rich BF (*n*)	Moderate BF (*n*)	Poor BF (*n*)	*p*
**Gender**				0.094
Male	112	102	12	
Female	96	56	6	
**Age (years)**				**0.015**
<45	32	40	6	
45–60	61	56	4	
>60	115	62	8	
**Marital status**				**0.021**
Married	180	111	14	
Unmarried	14	26	2	
Divorced	6	10	1	
Widowed	8	11	1	
**Education**				0.077
Primary or lower	6	4	1	
Junior high	52	51	2	
Senior high	84	42	6	
College or higher	66	61	9	
**Occupation**				0.075
Student	1	0	0	
Full-time	36	38	4	
Part-time	5	9	0	
Retire	148	88	10	
Unemployment	18	23	4	
**Economic burden**				0.279
Very mild	33	13	2	
Mild	58	42	4	
Moderate	84	71	6	
Serious	24	27	5	
Very serious	9	5	1	
**Number of chronic diseases**				**0.015**
0	48	23	4	
1	87	52	4	
2	51	44	8	
3	14	26	1	
4	4	8	0	
≥5	4	5	1	
**Duration of MHD**				0.351
0–1 year (including 1 year)	43	27	3	
1–5 years (including 5 years)	71	53	10	
5–10 years (including 10 years)	46	34	2	
10–20 years (including 20 years)	36	37	1	
>20 years	12	7	2	
**Kidney transplantation**				0.136
Yes	12	18	1	
No	196	140	17	
**Understanding degree of MHD**				**<0.001**
No understanding	40	15	0	
Partial understanding	97	43	3	
Adequate understanding	61	84	9	
Complete understanding	10	16	6	
**Self-assessed MHD severity**				**0.002**
Very mild	23	5	1	
Mild	82	40	6	
Moderate	102	108	11	
Serious	0	4	0	
Very serious	1	1	0	
**Self-assessed life impact**				0.539
Very mild	4	0	0	
Mild	25	20	3	
Moderate	110	78	7	
Serious	60	54	8	
Very serious	9	6	0	

The bold values indicates *p* < 0.05.

#### 3.4.2 Influences of social support and coping styles on the latent profile

**Figure 2:**
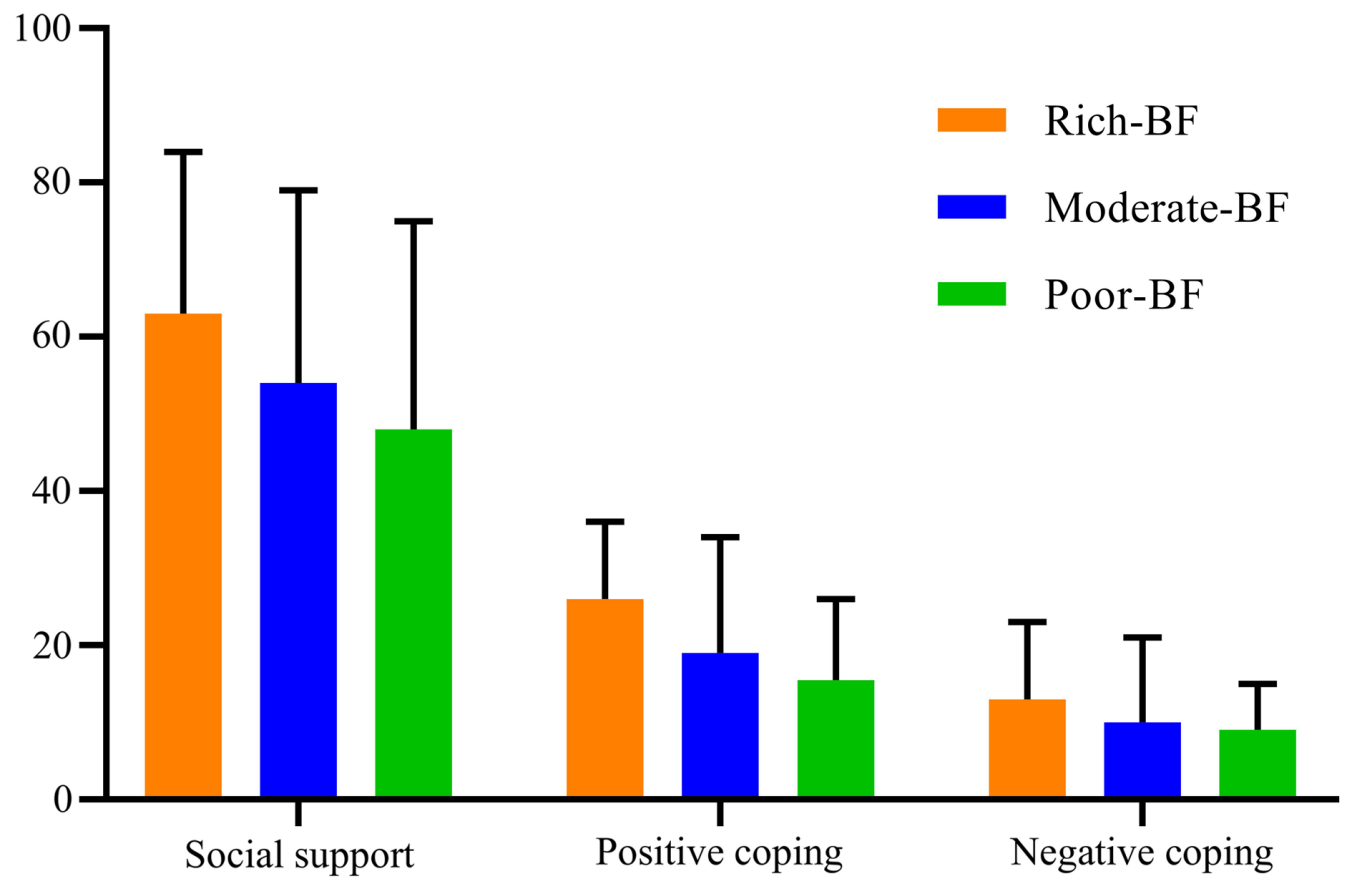
Conditional mean values of the three latent profiles on the social support and coping styles.

Differences were noted between BF scores, social support, and coping styles, as well as in the scores of each dimension (*p* < 0.05). No significant difference was observed in the duration of MHD (*p* > 0.05). The detailed results are shown in Table 6 and Figure 2.

**Table 6:** Comparison of the scores of social support coping style duration of MHD with different latent profiles.

Variable	Rich BF (M ± SD)	Moderate BF (M ± SD)	Poor BF (M ± SD)	*p*
Social support	62.68 ± 11.54	54.05 ± 11.26	51.28 ± 13.97	**<0.001**
Family support	24.31 ± 3.83	21.86 ± 4.49	18.89 ± 5.84	**<0.001**
Friend support	19.85 ± 5.51	16.07 ± 5.66	16.06 ± 7.33	**<0.001**
Other support	18.53 ± 6.26	16.12 ± 5.01	16.33 ± 4.97	**<0.001**
Positive coping	25.63 ± 6.43	18.70 ± 5.82	13.17 ± 8.60	**<0.001**
Negative coping	12.75 ± 4.42	10.47 ± 3.76	9.33 ± 3.48	**<0.001**
Duration of MHD	6.98 ± 6.84	8.29 ± 7.42	4.34 ± 2.99	0.682

The bold values indicates *p* < 0.05.

### 3.5 Analysis of the influencing factors of BF

A parallelism test conducted on the included variables yielded *p* = 0.802, meeting the standard criteria. An ordered multiple logistic regression analysis was conducted with the three latent profile groups as dependent variables and significant variables identified the univariate analysis as independent variables. The logistic model fitting was statistically significant (*χ*^2^ = 190.971, *p* < 0.001). Results indicated significant correlations of BF with *positive coping, social support, age,* and *understanding of MHD* (*p* < 0.05). Specifically, positive coping and social support emerged as protective factors for BF, with older age and enhanced MHD knowledge associated with higher BF levels. The detailed results are shown in Table 7.

**Table 7:** Results of multiple logistic regression analysis.

Independent variable	β	SE	Wald χ2	*p*	95% CI	
					Lower	Upper
Positive coping	−0.135	0.031	18.5	**<0.001**	−0.197	−0.074
Negative coping	−0.072	0.05	2.089	0.148	−0.171	0.026
Social support	−0.023	0.011	3.935	**0.047**	−0.045	0
**Age (years)**						
<45	1.087	0.387	7.89	**0.005**	0.329	1.846
45–60	0.61	0.297	4.23	**0.04**	0.029	1.192
>60						
**Marital status**						
Married	−0.422	0.585	0.52	0.471	−1.568	0.724
Unmarried	0.125	0.65	0.037	0.847	−1.148	1.399
Divorced	0.163	0.805	0.041	0.839	−1.415	1.741
**Widowed**						
Number of chronic diseases	−0.339	0.818	0.172	0.678	−1.941	1.263
0						
1	−0.572	0.796	0.516	0.472	−2.132	0.988
2	−0.097	0.791	0.015	0.902	−1.648	1.454
3	0.176	0.828	0.045	0.831	−1.446	1.798
4	−0.098	1.011	0.009	0.922	−2.08	1.883
≥5						
**Understanding degree of MHD**						
No understanding	−1.372	0.54	6.456	**0.011**	−2.431	−0.314
Partial understanding	−1.298	0.458	8.014	**0.005**	−2.196	−0.399
Adequate understanding	−0.337	0.435	0.598	0.439	−1.19	0.516
Complete understanding						
**Self-assessed MHD severity**						
Very mild	−0.024	1.649	0	0.988	−3.257	3.209
Mild	0.698	1.566	0.199	0.656	−2.371	3.767
Moderate	1.372	1.557	0.776	0.378	−1.68	4.424
Serious	1.653	1.879	0.775	0.379	−2.029	5.335
Very serious						
**Coping style**						
Positive coping	−0.333	0.414	0.645	0.422	−1.144	0.479
Negative coping						

The bold values indicates *p* < 0.05.

## 4 Discussion

This multi-center cross-sectional study investigated BF in 384 individuals undergoing MHD. Three distinct groups were identified as follows: rich BF, moderate BF, and poor BF. Overall, the findings emphasized the importance of recognizing these categories when exploring BF in renal care settings. Specifically, there is a need to sustain and enhance BF levels in the rich BF and moderate BF groups while providing targeted assistance to the poor BF group to address their specific challenges. Furthermore, we observed that older age, a deeper understanding of MHD, and higher levels of social support were correlated with higher BF levels. Both positive and negative coping styles were also associated with BF in this study sample.

### 4.1 Latent profile characteristics of BF among individuals on MHD

Previous research has indicated that BF levels in individuals undergoing MHD are generally lower than those with other chronic diseases (Dan-li and Wen-huan, 2022; Jue et al., 2023). However, these studies typically provide an overview of overall BF levels. This study represents a significant advancement by employing contemporary person-centered techniques to model individual characteristics (Çıtak, 2023). This approach enables the categorization of distinct BF groups among MHD individuals, paving the way for more targeted or personalized psychological nursing interventions. In terms of the number of individuals in different groups, there was significant heterogeneity in the BF of MHD individuals across six dimensions. The groups could be divided into rich BF (*n* = 208), moderate BF (*n* = 158), and poor BF (*n* = 18). The majority of individuals fall into the rich BF (54.17%) and moderate BF (41.15%) groups, indicating that they possess BF and may have a better ability to identify benefits following dialysis. However, it is important to acknowledge that a small number of individuals still exhibit extremely low levels of BF. Despite previous reports of poor psychological states in individuals on MHD, including a propensity for anxiety, depression, and other psychological problems (Elezi et al., 2023; Liu et al., 2024), this study recognizes the strong inner resilience in most of them (Wang et al., 2024). Most of the individuals have the ability to accept the negative events of dialysis and demonstrate positive psychology, i.e., they can find benefits (Li et al., 2018). Nonetheless, special attention should be paid to this small group with poor BF, identifying them through their emotional, linguistic, and behavioral traits in daily life and providing early psychological care, such as enhancing social support and guiding changes in coping styles (Wen et al., 2023).

In terms of BF scores, when compared with previous studies using the same research tools and methods (Ping et al., 2022), the latent profiles of BF for elderly individuals with chronic disease in the community were categorized as excellent benefit type (116.50 ± 3.50, *n* = 12), good benefit type (93.83 ± 6.45, *n* = 120), average benefit type (84.18 ± 5.95, *n* = 146), and low benefit type (65.49 ± 6.37, *n* = 35). The scores in this study were lower but more concentrated in population distribution, with fewer people scoring at the highest or lowest levels. This phenomenon could be attributed to differences in the demographic and disease characteristics of the participants. Studies have shown a positive correlation between age and BF (Wang et al., 2015; Lassmann et al., 2021), with younger individuals tending to have lower BF. The average age of participants in this study was lower (57.90 ± 13.36) than the aforementioned study, which consisted of individuals over 60 years of age. This finding aligns with our results where age is an influencing factor as supported by our regression analysis, showing that older MHD individuals have higher BF. Additionally, the specific characteristics of MHD, a lifelong and irreversible situation that individuals experienced in their lives with significant reductions in quality of life (Daniel et al., 2021), may also contribute to these differences. The requirement for individuals to undergo dialysis multiple times a week for several hours (National Kidney Foundation, 2015) imposes a significant adverse effect on daily life. Continuous MHD affects living habits, life planning, and family functions, leading to heightened physical burden (Daniel et al., 2021) and social pressure (Ma et al., 2021), which in turn can impair mental health (Chan et al., 2012).

### 4.2 Influences of demographic variables on the latent profile

While previous research has not consistently agreed on the relationship between demographic factors and BF, various studies have suggested correlations with gender, ethnicity, education level, marital status, and income level (Bi et al., 2021; Zimmaro et al., 2021; Wepf et al., 2022). In this study, the univariate analysis revealed that the BF in individuals on MHD across different latent profiles was associated with age, marital status, the number of chronic diseases, and the individual’s self-perceived knowledge and severity regarding MHD. Specifically, BF tended to be higher among those who were married, implying the positive influence of partner and family support in daily life. Consistent with other research (Sang et al., 2019), individuals with fewer chronic conditions exhibited higher BF levels, suggesting a link between better overall health and an increased capacity for BF. Additionally, the extent of an individual’s understanding of MHD and their perception of its severity significantly influenced their BF scores. Considerable awareness and comprehension of dialysis-related knowledge and disease symptoms correlated with higher BF scores. The regression analysis further underscored that age and knowledge about MHD were significant predictors of BF among the different groups. Older individuals, with their richer life experiences and enhanced ability to cope with negative events, were more likely to discern positive aspects in adverse environment, as indicated by their higher BF scores. This trend was most pronounced in those over 60 years old, intermediate in the 45–60 age group, and lowest in those under 45 years old. In terms of knowledge about MHD, comprehensive and sufficient understanding emerged as protective factors. This finding highlights the importance of providing patients with relevant disease information and nursing knowledge to enhance their disease management and confidence in combating their disease. Educational initiatives and decision aids are essential in improving understanding of MHD, fostering a sense of control over the condition. Additionally, early education has been linked to lower mortality rates after dialysis initiation (Chan et al., 2019).

### 4.3 Difference in the latent profiles of BF in coping style

In this study, the coping style refers to the cognitive and behavioral efforts employed by individuals to maintain psychological balance when confronted with stress, specifically hemodialysis (Folkman et al., 1986). The coping style is categorized into positive coping (e.g., problem-solving strategies like confrontation and seeking help) and negative coping (e.g., problem-avoidance strategies like withdrawal and endurance) (Huang et al., 2021; Lai et al., 2023). The Pearlin stress model highlights the coping style as a critical factor influencing health outcomes (Pearlin et al., 1990). The univariate analysis indicated that both positive and negative coping styles were associated with BF in different MHD groups. The regression analysis further revealed that positive coping was negatively correlated with BF across different categories, signifying its role as a protective factor for BF. This aspect aligns with previous studies (Bi et al., 2021). BF is the adaptive response of individuals to difficult situations that they encounter in their lives, and positive coping can promote patients to adapt to the adverse environment (Qiu et al., 2022), suggesting that positive coping facilitates an individual’s adaptation to adverse environments and is a key component in mitigating negative emotions (Miao et al., 2022). A semi-structured interview study suggested that healthier coping styles are likely to improve the physical condition of individuals with MHD, while unhealthy coping styles may exacerbate their physical burden (Wen et al., 2023). Additionally, positive coping styles have been found to activate individual potential, boost confidence in managing MHD, and foster a sense of psychological control (Al Sharji et al., 2022). Given that coping styles as emotional management strategies are difficult to modify (Lai et al., 2023), future initiatives aimed at promoting mental health in this population should focus on guiding individuals toward positive coping through interventions such as cognitive–behavioral therapy, meditation, relaxation techniques, and spiritual practices (Natale et al., 2019; Barello et al., 2023). These interventions have previously been suggested to facilitate more effective confrontation with adverse events and negative emotions in individuals on hemodialysis (Natale et al., 2019; Barello et al., 2023). Over time, such interventions can gradually influence the evolution of coping styles, leading to enhanced psychological resilience in the face of stress (Peng et al., 2022).

### 4.4 Difference in the latent profiles of BF in social support

This study reinforces the role of social support as a positive influence on BF in individuals with MHD, corroborating findings from previous research (Wang et al., 2015; Qiu et al., 2022). The regression analysis indicated that higher levels of social support correspond to increased BF. According to Pearlin’s stress model, social support is an important mediator of psychological stress, effectively alleviating the pressures experienced by individuals (Pearlin et al., 1990). Those reporting substantial positive social interactions often exhibit higher self-efficacy and access to resources for managing stress and illness burdens (Noviana and Zahra, 2021). A qualitative study highlighted how positive social interactions instill a sense of life purpose and future hope in individuals on MHD (Raj et al., 2020). It appears that among those experiencing long-term stress, individuals who employ positive and healthy coping styles often have diverse strategies for stress management (Labrague et al., 2018), enabling them to self-adjust and enhance their mental health status and quality of life (Zamanian et al., 2018). Social support plays a vital role in regulating mental health in this group. Semi-structured interviews revealed that support for individuals on MHD primarily originates from family members, friends, and medical staff (Wen et al., 2023). Individuals on MHD often receive substantial support from their families and relatives. In the context of their social networks, the family unit plays a crucial role. Family rearrangements, fostering intergenerational coexistence, are particularly significant (Santos et al., 2021). These familial interactions, along with friendships, can be a source of happiness for individuals on MHD. Additionally, medical staff primarily provide these individuals with medical support (Wen et al., 2023). However, the findings of this study highlight a notable aspect: When compared to family support, support from friends and others received lower scores. This discrepancy suggests that medical workers should offer more comprehensive emotional and informational support in their daily interactions with individuals on MHD (Chan et al., 2019). There is a need to enhance the families’ psychosocial understanding of hemodialysis and to develop coping skills for managing both the physical and emotional effects of the treatment. Setting future life goals and reinforcing personal and family identity are also important (Rolland, 2019). Engaging in these activities can empower individuals on MHD to boost their confidence, foster a sense of hope, and aid them in recognizing positive events and social resources. This approach can facilitate a more proactive attitude toward their condition and ultimately contribute to an improved level of BF (Griva et al., 2021).

### 4.5 Implications for practice and research

The findings of this study highlight the potential of utilizing BF as a central indicator in the development of health education and social–psychological intervention programs for individuals undergoing MHD (Griva et al., 2021). Embracing positive psychology as the guiding principle, these programs can be designed to engage patients in activities such as self-disclosure, simulation exercises, and group discussions. Such activities aim to fully harness an individual’ s positive resources throughout the hemodialysis process, encourage them to discover and harness their internal strengths, and foster positive cognitive and behavioral transformations. The abovementioned interventions have been partially applied in other populations and showed positive outcomes in improving BF. Antoni et al. (2001) and Cruess et al. (2000) were the first to conduct cognitive–behavioral stress management (CBSM) programs in individuals with breast cancer. The CBSM program consisted of stress management (e.g., cognitive restructuring and coping skills training), relaxation training (e.g., progressive muscle relaxation and meditation), and group discussions (personal experiences, experiential exercises, and role-playing), accompanied by homework exercises. The results found that CBSM can improve the level of benefit finding in patients. Zhang et al. (2021) conducted a study on 40 women with breast cancer who were randomized either to a guided self-disclosure intervention (GSDI) group, which included a six-session face-to-face self-disclosure intervention, or to a control group; the results showed that GSDI may be feasible in the clinic and might improve BF. Additionally, researchers have demonstrated that yoga (Chandwani et al., 2010) and progressive muscle relaxation combined with Chinese medicine five-element music can enhance the BF of individuals (Liao et al., 2018). These above approaches not only increase personal happiness but also contribute to improved long-term quality of life (Cruess et al., 2000; Antoni et al., 2001; Chandwani et al., 2010; Liao et al., 2018; Zhang et al., 2021). Finally, it is important to acknowledge the intrinsic value of interventions aimed at promoting psychological well-being, regardless of their direct impact on disease progression. For instance, even if a psychological intervention does not significantly reduce the frequency of MHD hospital visits (e.g., from 3 per week to 1 per week), its contribution to the overall mental health and quality of life of patients remains significant.

### 4.6 Strengths and limitations

One of the major strengths of this study is its pioneering exploration of latent BF profiles in individuals undergoing MHD. This study offers a novel comparison of these profiles in terms of general demographic characteristics and examines the correlations between BF groups and factors such as coping styles and social support.

However, there are some notable limitations to this study. Given its cross-sectional design and sample characteristics, the results and conclusions should be interpreted with caution. A key observation in the study was the uneven distribution of participants across BF groups, particularly the small number of individuals in the poor BF group (*n* = 18), which limits the statistical power of these findings. Future studies should validate our results in larger and more diverse samples. Despite this limitation, our study highlights the existence of a small but significant subset of individuals on MHD who exhibit low levels of BF and face substantial mental health challenges, necessitating prompt attention. Additionally, this study’s findings are influenced by its cultural context. Given our focus on a Mainland Chinese population, the applicability of the results to other communities may be limited due to cultural differences.

## 5 Conclusion

The present study showed the importance of exploring the different BF categories and their influencing factors in individuals on MHD, providing a theoretical ground for the development of subsequent intervention programs aimed at fostering BF in this population and, thus, improving the mental health and quality of life of individuals receiving MHD.

## Data availability statement

The raw data supporting the conclusions of this article will be made available by the authors, without undue reservation.

## Ethics statement

This study complied with the Declaration of Helsinki for studies with human participants and was approved by the Committee on Ethics of Medicine, Naval Medical University, PLA. Informed consent was obtained from the study subjects before the questionnaires were distributed.

## Author contributions

JY: Data curation, Formal analysis, Investigation, Methodology, Software, Supervision, Writing – original draft, Writing – review & editing. Y-qL: Data curation, Investigation, Methodology, Software, Writing – original draft, Writing – review & editing. Y-lG: Data curation, Investigation, Software, Writing – original draft. H-lY: Data curation, Investigation, Methodology, Writing – review & editing. JChe: Conceptualization, Project administration, Resources, Supervision, Writing – review & editing. L-lL: Project administration, Resources, Supervision, Writing – review & editing. JW: Conceptualization, Formal analysis, Methodology, Project administration, Supervision, Writing – review & editing. JChu: Conceptualization, Formal analysis, Funding acquisition, Methodology, Project administration, Supervision, Writing – review & editing.

## References

[ref1] Al Sharji A. Alaloul F. Al Y. B. (2022). Coping strategies in end-stage renal disease patients on hemodialysis in Oman: optimistic, supportive, Confrontive, and prayerful. J. Relig. Health 61, 2072–2082. doi: 10.1007/s10943-022-01579-5, PMID: 35576024

[ref2] Antoni M. H. Lehman J. M. Kilbourn K. M. Boyers A. E. Culver J. L. Alferi S. M.. (2001). Cognitive-behavioral stress management intervention decreases the prevalence of depression and enhances benefit finding among women under treatment for early-stage breast cancer. Health Psychol. 20, 20–32. doi: 10.1037//0278-6133.20.1.20, PMID: 11199062

[ref3] Aspinwall L. G. Tedeschi R. G. (2010). The value of positive psychology for health psychology: progress and pitfalls in examining the relation of positive phenomena to health. Ann. Behav. Med. 39, 4–15. doi: 10.1007/s12160-009-9153-0, PMID: 20091429

[ref4] Barello S. A.-O. Anderson G. Acampora M. Bosio C. Guida E. Irace V.. (2023). The effect of psychosocial interventions on depression, anxiety, and quality of life in hemodialysis patients: a systematic review and a meta-analysis. Int. Urol. Nephrol. 55, 897–912. doi: 10.1007/S11255-022-03374-3, PMID: 36180655 PMC10030538

[ref5] Bi W. Wang H. Yang G. Zhu C. (2021). A longitudinal cohort study on benefit finding evolution in Chinese women breast cancer survivals. Sci. Rep. 11:20640. doi: 10.1038/s41598-021-99809-5, PMID: 34667257 PMC8526563

[ref6] Chan C. T. Blankestijn P. J. Dember L. M. Gallieni M. Harris D. C. H. Lok C. E.. (2019). Dialysis initiation, modality choice, access, and prescription: conclusions from a kidney disease: improving global outcomes (KDIGO) controversies conference. Kidney Int. 96, 37–47. doi: 10.1016/j.kint.2019.01.017, PMID: 30987837

[ref7] Chan R. Brooks R. Steel Z. Heung T. Erlich J. Chow J.. (2012). The psychosocial correlates of quality of life in the dialysis population: a systematic review and meta-regression analysis. Qual. Life Res. 21, 563–580. doi: 10.1007/s11136-011-9973-9, PMID: 21805367

[ref8] Chandwani K. D. Thornton B. Perkins G. H. Arun B. Raghuram N. V. Nagendra H. R.. (2010). Yoga improves quality of life and benefit finding in women undergoing radiotherapy for breast cancer. J. Soc. Integr. Oncol. 8, 43–55. PMID: 20388445

[ref9] Çıtak Ş. (2023). Latent profile analysis of gambling. Front. Psychol. 14:1293933. doi: 10.3389/fpsyg.2023.1293933, PMID: 37965671 PMC10641010

[ref10] Cockwell P. Fisher L. A. (2020). The global burden of chronic kidney disease. Lancet 395, 662–664. doi: 10.1016/s0140-6736(19)32977-0 32061314

[ref11] Cruess D. G. Antoni M. H. McGregor B. A. Kilbourn K. M. Boyers A. E. Alferi S. M.. (2000). Cognitive-behavioral stress management reduces serum cortisol by enhancing benefit finding among women being treated for early stage breast cancer. Psychosom. Med. 62, 304–308. doi: 10.1097/00006842-200005000-00002, PMID: 10845343

[ref12] Daniel S. C. Azuero A. Gutierrez O. M. Heaton K. (2021). Examining the relationship between nutrition, quality of life, and depression in hemodialysis patients. Qual. Life Res. 30, 759–768. doi: 10.1007/s11136-020-02684-2, PMID: 33108580

[ref13] Dan-li P. Wen-huan H. (2022). Analysis of status quo and influencing factors of benefit finding in maintenance hemodialysis patients. J. Bengbu Med. Coll. 47, 122–125. doi: 10.13898/j.cnki.issn.1000-2200.2022.01.030

[ref14] de Vries E. F. Los J. de Wit G. A. Hakkaart-van R. L. (2021). Patient, family and productivity costs of end-stage renal disease in the Netherlands; exposing non-healthcare related costs. BMC Nephrol. 22:341. doi: 10.1186/s12882-021-02548-y, PMID: 34656083 PMC8520215

[ref15] Elezi B. Abazaj E. Zappacosta B. Hoxha M. (2023). Anxiety and depression in geriatric hemodialysis patients: factors that influence the border of diseases. Front. Psychol. 14:1281878. doi: 10.3389/fpsyg.2023.1281878, PMID: 38078242 PMC10704351

[ref16] Folkman S. Lazarus R. S. Gruen R. J. DeLongis A. (1986). Appraisal, coping, health status, and psychological symptoms. J. Pers. Soc. Psychol. 50, 571–579. doi: 10.1037//0022-3514.50.3.571 3701593

[ref17] GBD Chronic Kidney Disease Collaboration (2020). Global, regional, and national burden of chronic kidney disease, 1990–2017: a systematic analysis for the global burden of disease study 2017. Lancet 395, 709–733. doi: 10.1016/s0140-6736(20)30045-3, PMID: 32061315 PMC7049905

[ref18] Griva K. Chia J. M. X. Goh Z. Z. S. Wong Y. P. Loei J. Thach T. Q.. (2021). Effectiveness of a brief positive skills intervention to improve psychological adjustment in patients with end-stage kidney disease newly initiated on haemodialysis: protocol for a randomised controlled trial (HED-start). BMJ Open 11:e053588. doi: 10.1136/bmjopen-2021-053588, PMID: 34548369 PMC8458344

[ref19] Helgeson V. S. Reynolds K. A. Tomich P. L. (2006). A meta-analytic review of benefit finding and growth. J. Consult. Clin. Psychol. 74, 797–816. doi: 10.1037/0022-006x.74.5.797, PMID: 17032085

[ref20] Huang Y. Su X. Si M. Xiao W. Wang H. Wang W.. (2021). The impacts of coping style and perceived social support on the mental health of undergraduate students during the early phases of the COVID-19 pandemic in China: a multicenter survey. BMC Psychiatry 21:530. doi: 10.1186/s12888-021-03546-y, PMID: 34706690 PMC8549419

[ref21] Jue H. Yu F. Lijuan Z. Qin L. Rong L. Na Y.. (2023). Status of benefit finding in maintenance hemodialysis patients and its influencing factors. J. Chengdu Med. Coll. 18, 239–242. doi: 10.3969/j.issn.1674-2257.2023.02.022

[ref22] Kao Z. Lingyan Z. Qiming L. (2023). Research on the supervision path of medical insurance fund in the field of hemodialysis [J]. China Health Insur. 1, 64–69. doi: 10.19546/j.issn.1674-3830.2023.1.011

[ref23] Labrague L. J. McEnroe-Petitte D. M. Papathanasiou I. V. Edet O. B. Tsaras K. Leocadio M. C.. (2018). Stress and coping strategies among nursing students: an international study. J. Ment. Health 27, 402–408. doi: 10.1080/09638237.2017.1417552, PMID: 29261007

[ref24] Lai W. Li W. Guo L. Wang W. Xu K. Dou Q.. (2023). Association between bullying victimization, coping style, and mental health problems among Chinese adolescents. J. Affect. Disord. 324, 379–386. doi: 10.1016/j.jad.2022.12.080, PMID: 36587905

[ref25] Lassmann I. Dinkel A. Marten-Mittag B. Jahnen M. Schulwitz H. Gschwend J. E.. (2021). Benefit finding in long-term prostate cancer survivors. Support Care Cancer 29, 4451–4460. doi: 10.1007/s00520-020-05971-3, PMID: 33447865 PMC8236447

[ref26] Li T. Liu T. Han J. Zhang M. Li Z. Zhu Q.. (2018). The relationship among resilience, rumination and posttraumatic growth in hemodialysis patients in North China. Psychol Health Med. 23, 442–453. doi: 10.1080/13548506.2017.1384553 28984169

[ref27] Li C. Ure C. Zheng W. Zheng C. Liu J. Zhou C.. (2023). Listening to voices from multiple sources: a qualitative text analysis of the emotional experiences of women living with breast cancer in China. Front. Public Health 11:1114139. doi: 10.3389/fpubh.2023.1114139, PMID: 36817918 PMC9935709

[ref28] Li L. Zhong H. Y. Xiao T. Xiao R. H. Yang J. Li Y. L.. (2023). Association between self-disclosure and benefit finding of Chinese cancer patients caregivers: the mediation effect of coping styles. Support Care Cancer 31:684. doi: 10.1007/s00520-023-08158-8, PMID: 37945919

[ref29] Liao J. Wu Y. Zhao Y. Zhao Y. C. Zhang X. Zhao N.. (2018). Progressive muscle relaxation combined with Chinese medicine five-element music on depression for Cancer patients: a randomized controlled trial. Chin. J. Integr. Med. 24, 343–347. doi: 10.1007/s11655-017-2956-0, PMID: 28497396

[ref30] Liu H. H. Wu C. L. Chiang Y. C. Tsai K. H. Chu T. L. Hsiao Y. C. (2024). Religion and spiritual health in patients with and without depression receiving hemodialysis: a cross-sectional correlational study. J. Nurs. Res. 32:e309. doi: 10.1097/jnr.0000000000000592, PMID: 38190331

[ref31] Ma S. J. Wang W. J. Tang M. Chen H. Ding F. (2021). Mental health status and quality of life in patients with end-stage renal disease undergoing maintenance hemodialysis. Ann. Palliat. Med. 10, 6112–6121. doi: 10.21037/apm-20-2211, PMID: 34118836

[ref32] Mei Y. X. Xiang D. D. Zhang Z. X. Twumwaah Budu J. Lin B. L. Chen S. Y. (2023). Family function, self-efficacy, care hours per day, closeness and benefit finding among stroke caregivers in China: a moderated mediation model. J. Clin. Nurs. 32, 506–516. doi: 10.1111/jocn.16290, PMID: 35285125

[ref33] Miao M. Zheng L. Wen J. Jin S. Gan Y. (2022). Coping with coronavirus disease 2019: relationships between coping strategies, benefit finding and well-being. Stress. Health 38, 47–56. doi: 10.1002/smi.3072, PMID: 34057274 PMC8237076

[ref34] Natale P. Palmer S. C. Ruospo M. Saglimbene V. M. Rabindranath K. S. Strippoli G. F. (2019). Psychosocial interventions for preventing and treating depression in dialysis patients. Cochrane Database Syst. Rev. 12:CD004542. doi: 10.1002/14651858.CD004542.pub3, PMID: 31789430 PMC6886341

[ref35] National Kidney Foundation (2015). KDOQI clinical practice guideline for hemodialysis adequacy: 2015 update. Am. J. Kidney Dis. 66, 884–930. doi: 10.1053/j.ajkd.2015.07.015 26498416

[ref36] Noviana C. M. Zahra A. N. (2021). Social support and self-management among end-stage renal disease patients undergoing hemodialysis in Indonesia. J. Public Health Res. 11:2733. doi: 10.4081/jphr.2021.2733, PMID: 35238191 PMC8941312

[ref37] Pearlin L. I. Lieberman M. A. Menaghan E. G. Mullan J. T. (1981). The stress process. J. Health Soc. Behav. 22, 337–356. doi: 10.2307/2136676 7320473

[ref38] Pearlin L. I. Mullan J. T. Semple S. J. Skaff M. M. (1990). Caregiving and the stress process: an overview of concepts and their measures. Gerontologist 30, 583–594. doi: 10.1093/geront/30.5.583, PMID: 2276631

[ref39] Peng Y. Xu Y. Yue L. Chen F. Wang J. Sun G. (2023). Resilience in informal caregivers of patients with heart failure in China: exploring influencing factors and identifying the paths. Psychol. Res. Behav. Manag. 16, 1097–1107. doi: 10.2147/prbm.S405217, PMID: 37056465 PMC10086222

[ref40] Peng L. Ye Y. Wang L. Qiu W. Huang S. Wang L.. (2022). Chain mediation model of perceived stress, resilience, and social support on coping styles of Chinese patients on hemodialysis during COVID-19 pandemic lockdown. Med Sci Monit 28:e935300. doi: 10.12659/MSM.935300, PMID: 35288530 PMC8934009

[ref41] Ping C. Zhen Y. Huijun Z. (2022). Latent profile analysis and its influencing factors for benefit finding in elderly patients with chronic diseases in the community. Chin. Nurs. Res. 36, 4300–4305. doi: 10.12102/j.issn.1009-6493.2022.23.033

[ref42] Pop-Jordanova N. D. Polenakovic M. H. (2013). Psychological characteristics of patients treated by chronic maintenance hemodialysis. Int. J. Artif. Organs 36, 77–86. doi: 10.5301/ijao.5000188, PMID: 23335381

[ref43] Qiu X. Zhang K. Zhang Y. Sun L. (2022). Benefit finding and related factors of patients with early-stage Cancer in China. Int. J. Environ. Res. Public Health 19:4284. doi: 10.3390/ijerph19074284, PMID: 35409965 PMC8999120

[ref44] Raj R. Brown B. Ahuja K. Frandsen M. Jose M. (2020). Enabling good outcomes in older adults on dialysis: a qualitative study. BMC Nephrol. 21:28. doi: 10.1186/s12882-020-1695-1, PMID: 31996167 PMC6988330

[ref45] Ravaghi H. Behzadifar M. Behzadifar M. Taheri Mirghaed M. Aryankhesal A. Salemi M.. (2017). Prevalence of depression in hemodialysis patients in Iran: a systematic review and Meta-analysis. Iran. J. Kidney Dis. 11, 90–98. 28270640

[ref46] Rolland J. S.. The family, chronic illness, and disability: an integrated practice model. APA handbook of contemporary family psychology: applications and broad impact of family psychology, Vol 2. APA handbooks in psychology^®^. Washington, DC, US: American Psychological Association; (2019). p. 85–102.

[ref47] Saeed F. Sardar M. Rasheed K. Naseer R. Epstein R. M. Davison S. N.. (2020). Dialysis decision making and preferences for end-of-life care: perspectives of Pakistani patients receiving maintenance Dialysis. J. Pain Symptom Manag. 60, 336–345. doi: 10.1016/j.jpainsymman.2020.03.009, PMID: 32201311 PMC7375006

[ref48] Sang M. Leng Y. N. Lei M. J. Xiong S. Jin C. (2019). The current status and influencing factors of benefit finding among patients with type 2 diabetes mellitus. Chin. J. Nurs. 36, 6–10. doi: 10.3969/j.issn.1008-9993.2019.07.002

[ref49] Santos D. Pallone J. M. Manzini C. S. S. Zazzetta M. S. Orlandi F. S. (2021). Relationship between frailty, social support and family functionality of hemodialysis patients: a cross-sectional study. Sao Paulo Med. J. 139, 570–575. doi: 10.1590/1516-3180.2021.0089.R1.0904221, PMID: 34706049 PMC9634838

[ref50] Sun L. Liu K. Li X. Zhang Y. Huang Z. (2022). Benefit-finding experiences of cervical cancer survivors in rural Yunnan province, China: a qualitative study. Nurs. Open 9, 2637–2645. doi: 10.1002/nop2.962, PMID: 34120415 PMC9584485

[ref51] Tao Y. Liu T. Li P. Lv A. Zhuang K. Ni C. (2023). Self-management experiences of haemodialysis patients with self-regulatory fatigue: a phenomenological study. J. Adv. Nurs. 79, 2250–2258. doi: 10.1111/jan.15578, PMID: 36794672

[ref52] Thoits P. A. (2010). Stress and health: major findings and policy implications. J. Health Soc. Behav. 51, S41–S53. doi: 10.1177/0022146510383499, PMID: 20943582

[ref53] Thurlow J. S. Joshi M. Yan G. Norris K. C. Agodoa L. Y. Yuan C. M.. (2021). Global epidemiology of end-stage kidney disease and disparities in kidney replacement therapy. Am. J. Nephrol. 52, 98–107. doi: 10.1159/000514550, PMID: 33752206 PMC8057343

[ref54] Tian N. Chen N. Li P. K. (2021). Depression in dialysis. Curr. Opin. Nephrol. Hypertens. 30, 600–612. doi: 10.1097/mnh.0000000000000741 34456238

[ref55] Tran V. Wiebe D. J. Fortenberry K. T. Butler J. M. Berg C. A. (2011). Benefit finding, affective reactions to diabetes stress, and diabetes management among early adolescents. Health Psychol. 30, 212–219. doi: 10.1037/a0022378, PMID: 21401255 PMC3122964

[ref56] Wang Y. Qiu Y. Ren L. Jiang H. Chen M. Dong C. (2024). Social support, family resilience and psychological resilience among maintenance hemodialysis patients: a longitudinal study. BMC psychiatry 24:76. doi: 10.1186/s12888-024-05526-4 38279114 PMC10811847

[ref57] Wang Y. Zhu X. Yang Y. Yi J. Tang L. He J.. (2015). What factors are predictive of benefit finding in women treated for non-metastatic breast cancer? A prospective study. Psychooncology 24, 533–539. doi: 10.1002/pon.3685, PMID: 25288217

[ref58] Webster A. C. Nagler E. V. Morton R. L. Masson P. (2017). Chronic kidney disease. Lancet 389, 1238–1252. doi: 10.1016/s0140-6736(16)32064-5 27887750

[ref59] Wen J. Fang Y. Su Z. Cai J. Chen Z. (2023). Mental health and its influencing factors of maintenance hemodialysis patients: a semi-structured interview study. BMC Psychol. 11:84. doi: 10.1186/s40359-023-01109-2, PMID: 36978141 PMC10054072

[ref60] Wen Z. Xie J. Wang H. H. (2023). Principles, procedures and programs of latent class models. J. East China Norm. Univ. Educ. Sci. 41, 1–15. doi: 10.16382/j.cnki.1000-5560.2023.01.001

[ref61] Wepf H. Joseph S. Leu A. (2022). Benefit finding moderates the relationship between young carer experiences and mental well-being. Psychol. Health 37, 1270–1286. doi: 10.1080/08870446.2021.1941961, PMID: 34180332

[ref62] Yan H. Yang J. Luo C. Zhang L. Tian Y. Cui S.. (2023). Development and psychometric assessment of the benefit finding scale for Chinese older adults with chronic diseases. Res. Gerontol. Nurs. 16, 44–52. doi: 10.3928/19404921-20230105-03, PMID: 36692437

[ref63] Yu Y. Liu Z. W. Li T. X. Li Y. L. Xiao S. Y. Tebes J. K. (2020). Test of the stress process model of family caregivers of people living with schizophrenia in China. Soc. Sci. Med. 259:113113. doi: 10.1016/j.socscimed.2020.113113, PMID: 32646627

[ref64] Zamanian H. A.-O. Poorolajal J. A.-O. Taheri-Kharameh Z. A.-O. (2018). Relationship between stress coping strategies, psychological distress, and quality of life among hemodialysis patients. Perspect. Psychiatr. Care 54, 410–415. doi: 10.1111/ppc.12284, PMID: 29689625

[ref65] Zhang M. M. Chen J. J. Zhang T. Wang Q. L. Li H. P. (2021). Feasibility and effect of a guided self-disclosure intervention designed to facilitate benefit finding in breast cancer patients: a pilot study. Eur. J. Oncol. Nurs. 50:101879. doi: 10.1016/j.ejon.2020.101879, PMID: 33338740

[ref66] Zhu P. Chen C. Liu X. Gu W. Shang X. (2022). Factors associated with benefit finding and mental health of patients with cancer: a systematic review. Support Care Cancer 30, 6483–6496. doi: 10.1007/s00520-022-07032-3, PMID: 35391575

[ref67] Zimmaro L. A. Deng M. Handorf E. Fang C. Y. Denlinger C. S. Reese J. B. (2021). Understanding benefit finding among patients with colorectal cancer: a longitudinal study. Support Care Cancer 29, 2355–2362. doi: 10.1007/s00520-020-05758-6, PMID: 32918129 PMC7947025

